# Silver as an Antiseptic, from a Surgical and Therapeutic Viewpoint

**Published:** 1897-08

**Authors:** 


### Silver as an Antiseptic, from a Surgical and Therapeutic Viewpoint.

Dr. Rieffel, in *Rev. de Chir.* (No. 1, p. 50, Jan. 10, 1897), gives some account of Crede's (of Dresden) contribution to the transactions of the twenty-fifth Congress of the German Surgical Society on this subject.—*Centralb. f. Chirurg.* (No. 31, 1896). Crede, accepting silver as a powerful antiseptic on the authority of Behring, Koch, Miller, etc., made use of gauze impregnated with silver solution only to find that, when the wounds were very infectious and secreted freely, the silver soon disappeared and the sterilizing action ceased. The silver was attacked by chemicals formed in the tissues, e. g., lactic acid which, uniting with it, formed a soluble lactate of higher germicide power. But, this lactate (actol of commerce), in contact with wounds, very often irritated them and caused intolerable pain, due to its great solubility (1 in 15). After trials, Crede settled upon the citrate (itrol) which up to the present has given excellent results. It is soluble in 3800 parts of water, is colorless, inodorless, non-irritating, non-toxic, easily pulverizable, and keeps well. Its antiseptic value is equal to that of lactate of silver.

Either an old or a recent wound is powdered with the itrol, and dressed with itrol gauze to remain undisturbed for eight days if needed, with absolutely no reaction and with a minimum of secretion. Finally he insists, on the incontestable value of hypodermic injections of actol in acure infections and especially in erysipelas.

Lauenstein (Hamburg) confirmed Crede's claims, and valued them the more because of their clinical proving. He had employed sutures impregnated with silver, and no longer found suppurations at the point of the sutures. He also disinfects the skin with silver before operation. In 147 cases in spite of the presence of the white staphylococcus, primary union was possible in the majority of cases.

Kœlliker (of Leipzig) lauds iodoform, combined with a derivative of formaldehyd, because sterilizable at 140° C., inodorous and non-irritating.—*U. in American Medico-Surgical Bulletin.*

